# Clinical impact of food processing

**DOI:** 10.1186/2045-7022-1-S1-S53

**Published:** 2011-08-12

**Authors:** Joseph Baumert

**Affiliations:** 1 University of Nebraska-Lincoln, Food Allergy Research and Resource Program, Department of Food Science & Technology, Lincoln, USA

## 

Food processing unit operations can have effects on food allergens and their capacity to provoke reactions. The vast majority of food allergens are proteins, although most individual proteins in foods do not possess allergenic activity. Thus, the physical removal of proteins e.g. oil refining can eliminate the allergenic activity as is well documented for highly refined peanut oil. Furthermore, the chemical and/or enzymatic hydrolytic destruction of proteins can dramatically decrease allergen risk as evidenced by the use of extensively hydrolyzed casein, a major milk allergen, in hypoallergenic infant formula intended for milk-allergic infants. However, since exposure to trace amounts of allergenic proteins can provoke reactions in some allergic individuals, the efficacy of these unit operations in eliminating allergen risk is of considerable concern. The assessment of the residual allergenicity of processed foods can be difficult. The methods employed in such assessments must yield results that can predict allergenicity upon ingestion of the processed food. Clearly, blinded oral challenges on allergic individuals are the ultimate approach to document the safety of a particular processed food product. However, this approach is arduous, expensive and potentially risky. Because the allergenic activity of a protein depends upon its ability to bind to specific IgE antibodies from the blood sera of allergic individuals, the effect of processing on the IgE-binding capacity of an extract of the processed food may offer some indication of its allergenic potential. However, IgE binding is often assessed by immunoassay, an approach that depends upon the solubility of the processing-modified protein. Processing can diminish the solubility of proteins and thereby decrease apparent IgE binding in immunoassays. But the possibility exists that insoluble allergens may remain allergenic after ingestion. A full understanding of the effects of processing on allergenicity, the specific allergens, and detection methods does not exist for any allergenic food.

